# First case of a naturally acquired human infection with *Plasmodium cynomolgi*

**DOI:** 10.1186/1475-2875-13-68

**Published:** 2014-02-24

**Authors:** Thuy H Ta, Shamilah Hisam, Marta Lanza, Adela I Jiram, NorParina Ismail, José M Rubio

**Affiliations:** 1 Malaria & Emerging Parasitic Diseases Laboratory, Parasitology Department, National Microbiology Centre, Instituto de Salud Carlos III, Cra. Majadahonda Pozuelo Km. 2, Majadahonda 28220, Madrid, Spain; 2 Paal Research, Jalan Pahang, Kuala Lumpur 50588, Malaysia

**Keywords:** *Plasmodium vivax*, *Plasmodium cynomolgi*, Molecular methods, Malaysia, Simian malaria

## Abstract

Since 1960, a total of seven species of monkey malaria have been reported as transmissible to man by mosquito bite: *Plasmodium cynomolgi, Plasmodium brasilianum*, *Plasmodium eylesi, Plasmodium knowlesi*, *Plasmodium inui*, *Plasmodium schwetzi* and *Plasmodium simium*. With the exception of *P. knowlesi*, none of the other species has been found to infect humans in nature. In this report, it is described the first known case of a naturally acquired *P. cynomolgi* malaria in humans.

The patient was a 39-year-old woman from a malaria-free area with no previous history of malaria or travel to endemic areas. Initially, malaria was diagnosed and identified as *Plasmodium malariae*/*P. knowlesi* by microscopy in the Terengganu State Health Department. Thick and thin blood films stained with 10% Giemsa were performed for microscopy examination. Molecular species identification was performed at the Institute for Medical Research (IMR, Malaysia) and in the Malaria & Emerging Parasitic Diseases Laboratory (MAPELAB, Spain) using different nested PCR methods.

Microscopic re-examination in the IMR showed characteristics of *Plasmodium vivax* and was confirmed by a nested PCR assay developed by Snounou *et al*. Instead, a different PCR assay plus sequencing performed at the MAPELAB confirmed that the patient was infected with *P. cynomolgi* and not with *P. vivax*.

This is the first report of human *P. cynomolgi* infection acquired in a natural way, but there might be more undiagnosed or misdiagnosed cases, since *P. cynomolgi* is morphologically indistinguishable from *P. vivax,* and one of the most used PCR methods for malaria infection detection may identify a *P. cynomolgi* infection as *P. vivax*.

Simian *Plasmodium* species may routinely infect humans in Southeast Asia. New diagnostic methods are necessary to distinguish between the human and monkey malaria species. Further epidemiological studies, incriminating also the mosquito vector(s), must be performed to know the relevance of cynomolgi malaria and its implication on human public health and in the control of human malaria.

The zoonotic malaria cannot be ignored in view of increasing interactions between man and wild animals in the process of urbanization.

## Background

It has been asked whether animal malaria could be considered as true zoonoses. It has been known for some time that malaria of some non-human primates may infect man [1]. The infectivity of the simian malaria parasites in humans through anopheline mosquitoes has been demonstrated experimentally [2,3], although natural transmission of a non-human *Plasmodium* species to humans was, until recently, thought to be rare [4]. In 1965, the first case of a naturally acquired *Plasmodium knowlesi* infection was described [5], but it was not until 2004 when Singh *et al*. [6] identified 120 (57.7%) individuals with malaria as single or mixed *Plasmodium knowlesi* infections, that simian malaria started to be considered as a concern for human public health.

The absence of confirmed naturally acquired infections of other simian species of *Plasmodium* should not discourage consideration and investigation of them as zoonosis [7]. Since 1960, a total of seven species of monkey malaria have been reported as transmissible to man by mosquito bite: *Plasmodium cynomolgi*[8-10]*, Plasmodium brasilianum*[11], *Plasmodium eylesi, Plasmodium knowlesi*[2,12], *Plasmodium inui*[13], *Plasmodium schwetzi*[14], and *Plasmodium simium*[15].

*Plasmodium cynomolgi*, *P. knowlesi*, *P. inui* and *P. eylesi* are distributed in Asia, *P. brasilianum* and *P. simium* in the New World, while *P. schwetzi* is found in Africa [1]. With the exception of *P. knowlesi*, none of the other species has been found to infect humans in nature.

Natural *P. knowlesi* infection may progress as a self-limiting malaria with spontaneous cure [12], although generally the presentation progresses as moderate or can be severe [4,16-19], requiring anti-malarial therapy [20-24]. The striking feature of induced cynomolgi malaria infections in man was the high degree of clinical manifestations observed in relation to the low level of parasitaemia [25]. Clinical symptoms consisted of cephalgia, anorexia, myalgia, and nausea, in that order. The symptoms were usually present only during febrile episodes, were of moderate severity, and easily controlled by simple medications. The most prominent physical findings were splenomegaly and hepatomegaly [25].

*Plasmodium cynomolgi* was first described by Mayer in 1907 in Germany in *Macaca cynomolgus* (= *Macaca fascicularis*) imported from Java [25]. The morphological features of *P. cynomolgi*, by microscopy, are almost identical to that of *Plasmodium vivax*. The host red blood cell enlarges as the trophozoite grows, accompanied by prominence of Schüffner’s stippling and pigmentations in mature trophozoites. The cytoplasm becomes amoeboid and pigments in small granules with yellowish brown in colour are scattered throughout the cytoplasm. The asexual cycle is completed in 48 hours. The prepatent periods range from seven to 16 days with a mean of 9.8 days. Similarly to *P. vivax*, *P. cynomolgi* presents hypnozoites, which can initiate relapses [25].

In this report, the first known case of naturally acquired *P. cynomolgi* malaria in humans is described.

### Case presentation

The patient was a 39-year-old Malay woman from the east coast of Peninsular Malaysia (Hulu Terengganu) with no previous history of malaria and an uneventful medical history. She lives in a modern housing area and behind her house there is a small, forested area with occasional sightings of long-tailed macaques. She works as a government nurse in a malaria-free area.

She experienced episodes of mild to moderate 24-hour cycles of morning fevers since 10 January, 2011 with chills and rigor, cough and cold. Four days prior, she visited her mother’s house, in a non-endemic malaria area (6–9 January, 2011) and did not travel to any other malaria-endemic areas. The symptoms were non-specific and mimicked a flu-like syndrome. She took two days of medical leave (13, 14 January) without seeking hospital treatment. She felt more ill and was still febrile after two weeks and sought treatment at the Terengganu State Health Department. She was admitted to hospital on 20 January. A series of blood films for malaria parasites were taken and screened by microscopy and an initial diagnosis of *Plasmodium malariae*/*P. knowlesi* was concluded. The Giemsa-stained thick blood smears revealed 0.024% *Plasmodium* parasitaemia (1,200 parasites/μl) with only asexual stages. She was given a three-day course of oral chloroquine (day 1: 600 mg base and 300 mg base six hours later; day 2 and day 3: 300 mg base daily, in total 25 mg base/kg), according to the Malaysian National Antibiotic Guidelines on management of malaria. The patient recovered and was discharged one week later (26 January, 2011). Microscopic examinations of her blood smears were performed weekly for the first month from discharge followed by monthly examinations for one year. All blood smears were negative.

The Malaysian Vector Borne Disease Control Programme team did a case investigation among residents within the patient’s housing area and surveyed the area for possible vectors. No other persons were infected and anopheline mosquitoes caught were negative for malaria parasites, as determined by mosquito dissection. In this area *Anopheles cracens* was the predominant mosquito which is the vector of *P. inui* and *P. cynomolgi*[26].

Patient blood samples were sent to the Institute for Medical Research (IMR, Kuala Lumpur, Malaysia) for further species confirmation by microscopy and molecular diagnostic methods. Microscopic re-examination of blood film showed characteristics of *P. vivax* rather than *P. malariae*/*P. knowlesi*, scanty trophozoites, larger than the size of *Plasmodium falciparum*, were visible in the “ghost” of red blood cells, with large chromatin dot and thick cytoplasm. Molecular species identification was performed by a nested PCR assay based on the small subunit (SSU) rRNA genes with primers specific for *P. vivax*, *P. falciparum*, *P. malariae, Plasmodium ovale* (Snounou-PCR) [27] and primers specific for *P. knowlesi* (Singh-PCR) [6]. Molecular diagnosis by the Snounou-PCR assay identified a *P. vivax* infection yielding the expected size fragment using the specific-primer pair for *P. vivax*; while the rest of the species-specific nested PCR assays for *P. malariae*, *P. falciparum* and the Singh-PCR for *P. knowlesi* did not produce any amplifications.

A DNA sample was sent to the Malaria & Emerging Parasitic Diseases Laboratory (MAPELAB, CNM-ISCIII, Madrid, Spain) as part of an informal quality control exchange. The sample was analysed by a modification of the nested multiplex malaria PCR (NM-PCR), also based on the small subunit (SSU) rRNA genes [28,29], adding a parallel nested PCR for *Plasmodium* genus amplification (NG-PCR). The NM-PCR method is able to identify the four human malaria species (*P. vivax*, *P. falciparum*, *P. ovale* and *P. malariae*) in two consecutive multiplexing amplifications, while the NG-PCR identifies all *Plasmodium* species. Primer sequences, concentration and annealing temperature to set up these methods are described in Table 1.

**Table 1:** Description of NM-PCR and NG-PCR with the corresponding primers sequences, concentration and annealing temperature

**Reaction**	**Primer name**	**Primer (μmol/L)**	**Sequence (5’-3’)**	**Annealing temp. (°C)**	**Product size (bp)**	**Specificity**
First PCR*	UNR	0.10	GACGGTATCTGATCGTCTTC	58°C		Universal
	PLF	0.10	AGTGTGTATCCAATCGAGTTTC		783–821§	*Plasmodium*
	HUF	0.01	GAGCCGCCTGGATACCGC		231	Human
NM-PCR†	NewPLFshort	0.15	CTATCAGCTTTTGATGTTAG	53°C		*Plasmodium*
	MARshort	0.10	TCCAATTGCCTTCTG		241	*P. malariae*
	FARshort	0.15	GTTCCCCTAGAATAGTTACA		370	*P. falciparum*
	OVRshort,	0.10	AGGAATGCAAAGARCAG		407	*P. ovale*
	VIRshort,	0.10	AAGGACTTCCAAGCC		476	*P. vivax*
NG-PCR‡	NewPLFshort	0.50	CTATCAGCTTTTGATGTTAG	53°C	735–773¶	*Plasmodium*
	NewRevshort	0.50	CCTTAACTTTCGTTCTTG			*Plasmodium*

*First PCR performed at final volume of 50 μl and 5 μl DNA template.

†Second multiplex PCR performed at final volume of 25 μl with 2 μl template from first PCR product.

‡Second genus PCR performed at final volume of 50 μl with 2 μl template from first PCR amplification.

§Size depending on *Plasmodium* species: *P. malariae* 821 bp, *P. falciparum* 787 bp, *P. vivax* 783 bp, *P. ovale* 794 bp, *P. knowlesi* 807 bp, *P. cynomolgi* 785 bp.

¶Size depending on *Plasmodium* species: *P. malariae* 773 bp, *P. falciparum* 739 bp, *P. vivax* 735 bp, *P. ovale* 746 bp, *P. knowlesi* 763 bp. *P. cynomolgi* 738 bp.

In the MAPELAB molecular diagnosis by NM-PCR was negative for the four human malaria species and positive for NG-PCR showing an amplified fragment of expected size, confirming that the patient indeed had malaria infection. Sequencing of amplified products, using PLF and UNR primers from the NM-PCR assay, were performed after DNA purification (Illustra DNA and Gel Band Purification Kit, General Electric Healthcare) by Cycle Sequencing using Big Dye Terminator v3.1 in an ABI PRISM® 3700 DNA Analyzer. The sequence obtained has been submitted to the GenBank data base with the accession number JQ794445.

**Figure 1:**
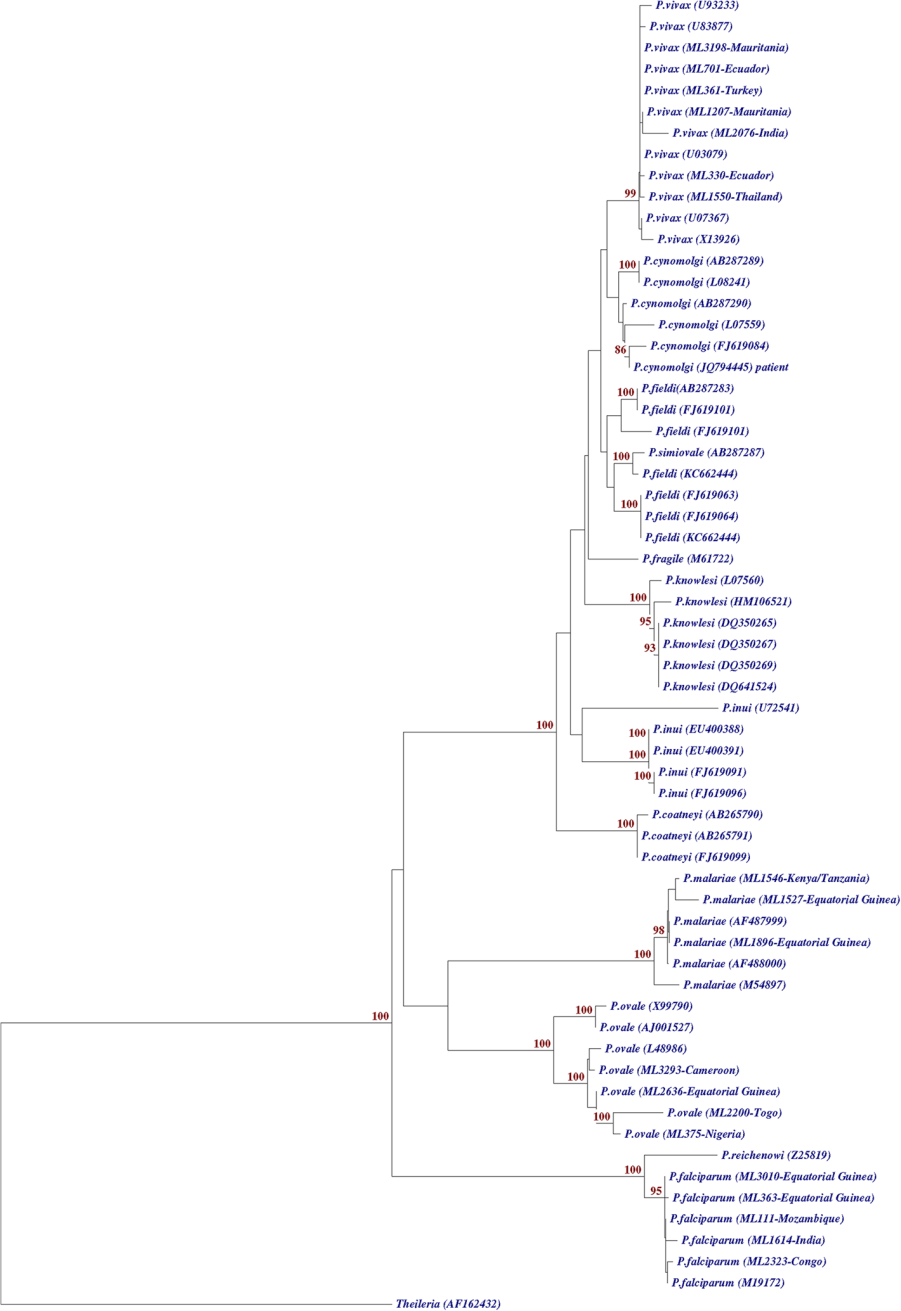
**Neighbour joining phylogenetic tree.** Phylogenetic tree comparing the A-type SSU rRNA gene sequence obtained (GenBank accession number JQ794445) with other *Plasmodium* species identified in our laboratory as well, but not submitted to the GenBank (identified as “ML plus an internal number” given in our Malaria Laboratory followed of the country name in the cases where this is known) and with known *Plasmodium* A-type SSU rRNA sequences from GenBank (accession numbers are indicated in parenthesis). The sequence of our patient clusters with all other *P. cynomolgi* strains. Sequence *Theileria* (AF162432) was used as outgroup. Significative bootstrap values are indicated.

A BLAST search of the sequence of 785 nucleotides in GenBank showed highest similarity with *P. cynomolgi* sequences, 99.9% with *P. cynomolgi* Mulligan strain from Malaysia (Accession number AB287290), followed by similarities between 99.6 and 99.4% with three other *P. cynomolgi* SSU rRNA sequences transcribed during the asexual stages (Accession No AB287289, L07559 and L08241), thereby confirming that the patient was infected with *P. cynomolgi*. Furthermore, a phylogenetic tree was performed, with Treecon software [30], after ClustalW alignment of the sequences [31] by Neighbour joining method, comparing the A-type SSU rRNA sequence obtained from the patient with sixty sequences from twelve *Plasmodium* species. The phylogenetic tree shows that patient’s sequence formed a monophyletic clade with *P. cynomolgi* independent of the clade formed for *P. vivax* sequences, adding another evidence that patient was infected with *P. cynomolgi* (Figure 1). In Madrid’s laboratory, no sample of *P. cynomolgi* had ever been present, so the result could not be caused by DNA contamination.

Morphologically, microscopic analysis of asexual stages of *P. cynomolgi* could be misidentified as *P. vivax*, because they are indistinguishable, and therefore, any infection in humans will be characterized as *P. vivax*[25]. In these cases, molecular diagnostic methods are the most accurate way to distinguish between species that are morphologically identical such as *P. knowlesi*/*P. malariae* or *P. vivax/P. cynomolgi*[4,5,11,25]. In this study, two different PCR methods gave discrepant results but sequencing showed an infection by *P. cynomolgi*.

NewPLFshort primer coupled to the specific *P. vivax* primer VIRsh in the NM-PCR did not show any amplification product as it was expected in a *P. cynomolgi* infection. In contrast, using the rVIV1 and rVIV2 primers, in the Snounou-PCR assay the patient sample was identified as *P. vivax* showing a possible cross amplification*.*

**Figure 2:**
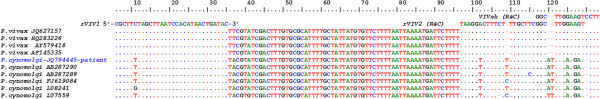
**Partial A-type SSU rRNA gene alignment from *Plasmodium vivax *and *Plasmodium cynomolgi *in vivax-primers regions.** Alignment of partial sequences from *Plasmodium vivax* and *Plasmodium cynomolgi* with the Malay case (in blue color and with the GenBank accession number JQ794445) taking as reference the primers VIRsh and rVIV1-rVIV2. Note: The alignment is in forward sense 5’-3’ and the VIVsh and rVIV2 primers sequences are in reverse and complementary form (R&C) to allow the correct alignment. (.) Represents an identical nucleotide. (-) Represents an alignment gap.

In the NM-PCR, NewPLFshort primer has 100% identity with *P. cynomolgi* in the corresponding region, since this primer is designed as a universal *Plasmodium* primer. Instead, VIRsh primer mismatches six nucleotides out of 15 (40%), including a gap and a double nucleotide insertion, in the corresponding region of the *P. cynomolgi* sequence (Figure 2). This primer has a melting temperature of 52.7°C and the PCR is performed at very stringent conditions of 53°C. On the other hand, comparing the Snounou primer sequence corresponding to rVIV1 primer with *P. cynomolgi* sequences, there is only one mismatch in the 30 nucleotides of the primer and the sequence of the rVIV2 primer shows seven mismatches out of 30 nucleotides (23.33%) including two gaps (one of 1 bp, another of 2 bp), most of them localized in the 5’-end primer region and just two in the 3’-end (Figure 2). Furthermore, the melting temperature of these primers is 66.3°C for rVIV1 and 73.2°C for rVIV2 [32], while the PCR is performed at 58°C [27,33], therefore a perfect alignment is not necessary and a *P. cynomolgi* amplification is very possible. The nested PCR assay developed by Snounou and co-workers has been widely used in Southeast Asia in numerous studies with great success, as evidenced by the literature [33-35]. However, based on the experience of this case and according to the primer alignment performed, it is very possible that vivax primers designed (rVIV1-rVIV2) could cross react with *P. cynomolgi* leading to false positive results for *P. vivax*.

Malaysia is a country endemic for malaria, particularly in the forested, hilly and underdeveloped interior areas of Malaysian Borneo (Sabah and Sarawak states) and Peninsular Malaysia. There is no risk in urban, suburban or coastal areas, including the east coast where the patient lives. Since the Malaria Eradication Programme started in the states of Sabah and Sarawak in 1961 [36] and in Peninsular Malaysia in 1967, malaria cases have dropped drastically over the years [37]. The major *Plasmodium* species are *P. vivax* (70%) and *P. falciparum* (30%) [38]. In some areas zoonotic malaria cases by *P. knowlesi,* morphologically indistinguishable from *P. malariae*, are frequent [6].

The symptoms of the patient were non-specific, she experienced episodes of mild to moderate 24-hour cycles of morning fevers with chills and rigor, cough and cold which mimicked a flu-like syndrome. These clinical symptoms were similar to those described in non-natural infection in humans and were usually present only during febrile episodes. These last experimental infections were performed with laboratory strains, which can be less virulent than natural strains [25]. Although patient experienced fever at 24 hourly intervals, when it was expected every 48 hours, like asexual cycle period of *P. cynomolgi*, previous reports of infected volunteers with *P. cynomolgi* showed that fever had different presentations, from afebrile to daily or tertian fever [3,25].

Despite she lives in a malaria-free area, physicians suspected malaria because the patient experienced the characteristic cyclic fever with chills. A malaria infection by *P. malariae/P. knowlesi* was the final diagnosis in the hospital and patient was treated with chloroquine, the fever and symptoms resolved and did not show any complications and no relapse at the time of writing this report. Treatment with primaquine to eliminate hypnozoite forms, characteristic of *P. vivax* and *P. cynomolgi*, was not prescribed as patient was diagnosed as *P. malariae/P. knowlesi*. Infections produced in many volunteers by mosquito bite in the 1950s to 1970s with *P. cynomolgi* sporozoite showed symptoms of moderate severity which were easily controlled by simple medications or even without treatment [25]. In these last volunteers no relapses were described which could indicate that hypnozoites of *P. cynomolgi* in humans are not activated to become hepatic schizonts, which suggests that a specific treatment with primaquine is not necessary to avoid relapses.

## Conclusions

This report describes the first known case of naturally acquired *P. cynomolgi* malaria in humans.

Morphologically, *P. cynomolgi* is indistinguishable from *P. vivax*, and as shown in this report, one of the most used PCR for malaria infection can characterize those infections as *P. vivax*. Microscopic examination of peripheral blood by thick and thin film is the golden standard for malaria diagnosis and species identification. Despite the high PCR sensitivity and specificity to detect malaria parasites in the blood, it has not been established as a routine diagnostic method in reference central laboratories to correct misdiagnosis in species identification, as well as increasing sensitivity in cases of individuals with low parasitaemia or asymptomatic carriers. As shown in this report and others, misdiagnosis in species identification can be more frequent than expected, especially in relation to non-human malaria, such as *P. knowlesi* or *P. cynomolgi*[4-6,25], as well as human infections involving *P. vivax*, *P. falciparum* and *P. knowlesi*[39].

Simian *Plasmodium* species could routinely infect humans in Southeast Asia and the correct diagnosis could be missed since it is not possible by microscopy to accurately identify *Plasmodium* species with similar morphology, such as that observed between *P. cynomolgi* and *P. vivax*. The application of new diagnostic methods is necessary to distinguish between the human and monkey malaria species. Further epidemiological studies, incriminating the mosquito vector(s), must be performed to know the relevance of cynomolgi malaria and its implication on human public health and in the control of human malaria.

The importance of zoonotic malaria transmissible by non-human primates cannot be ignored in view of increasing interactions between humans and the wild animals in the process of urbanization.

### Consent

Oral and written informed consent was obtained from the patient for publication of this case report and any accompanying images after explanation of the report objectives.

## Abbreviations

Bp: Basepairs; DNA: Deoxyribonucleic acid; IMR: Institute for medical research; MAPELAB: Malaria & emerging parasitic diseases laboratory; NG-PCR: Nested *Plasmodium* genus PCR; NM-PCR: Nested multiplex malaria PCR; PCR: Polymerase chain reaction; SSU rRNA: Small subunit ribosomal ribonucleic acid.

## Competing interests

The authors declare that they have no competing interests.

## Authors’ contributions

JMR supervised molecular characterization of parasite, conceived the study, its design and coordination, analysed the data and corrected the manuscript. THT carried out the molecular methods (NM-PCR and NG-PCR), analysed the sequence alignment, the data and drafted the manuscript. SH participated in the design and coordination of the study, and drafted the manuscript. AIJ and NI performed the patient DNA isolation, stained and examined the slides by microscopy and carried out the molecular methods (Snounou-PCR and Singh-PCR assays). ML was the responsible the sequencing and archive blood sample. All authors read and approved the final manuscript.

## References

[B1] Contacos PG Primate malarias: man and monkeys J Wildl Dis 1970 6 323 328 10.7589/0090-3558-6.4.323 16512134

[B2] Chin W Contacos PG Collins WE Jeter MH Alpert E Experimental mosquito-transmission of *Plasmodium knowlesi* to man and monkey Am J Trop Med Hyg 1968 17 355 358 4385130 10.4269/ajtmh.1968.17.355

[B3] Contacos PG Elder HA Coatney GR Genther C Man to man transfer of two strains of *Plasmodium cynomolgi* by mosquito bite Am J Trop Med Hyg 1962 11 186 193 13880974 10.4269/ajtmh.1962.11.186

[B4] Kantele A Jokiranta TS Review of cases with the emerging fifth human malaria parasite, *Plasmodium knowlesi* Clin Infect Dis 2011 52 1356 1362 10.1093/cid/cir180 21596677

[B5] Chin W Contacos PG Coatney GR Kimball HR A naturally acquired quotidian-type malaria in man transferable to monkeys Science 1965 149 865 14332847 10.1126/science.149.3686.865

[B6] Singh B Kim SL Matusop A Radhakrishnan A Shamsul SS Cox-Singh J Thomas A Conway DJ A large focus of naturally acquired *Plasmodium knowlesi* infections in human beings Lancet 2004 363 1017 1024 10.1016/S0140-6736(04)15836-4 15051281

[B7] Baird JK Malaria zoonoses Travel Med Infect Dis 2009 7 269 277 10.1016/j.tmaid.2009.06.004 19747661

[B8] Coatney GR Elder HA Contacos PG Getz ME Greenland R Rossan RN Schmidt LH Transmission of the M strain of *Plasmodium cynomolgi* to man Am J Trop Med Hyg 1961 10 673 678 13694174 10.4269/ajtmh.1961.10.673

[B9] Eyles DE Coatney GR Getz ME *Vivax*-type malaria parasite of macaques transmissible to man Science 1960 131 1812 1813 10.1126/science.131.3416.1812 13821129

[B10] Schmidt LH Greenland R Genther CS The transmission of *Plasmodium cynomolgi* to man Am J Trop Med Hyg 1961 10 679 688 13748031 10.4269/ajtmh.1961.10.679

[B11] Contacos PG Lunn JS Coatney GR Kilpatrick JW Jones FE Quartan-type malaria parasite of new world monkeys transmissible to man Science 1963 142 676 14068213 10.1126/science.142.3593.676

[B12] Ta TT Salas A Ali-Tammam M Martinez MC Lanza M Arroyo E Rubio JM First case of detection of *Plasmodium knowlesi* in Spain by Real Time PCR in a traveller from Southeast Asia Malar J 2010 9 219 10.1186/1475-2875-9-219 20663184 PMC2921078

[B13] Coatney GR Chin W Contacos PG King HK *Plasmodium inui*, a quartan-type malaria parasite of Old World monkeys transmissible to man J Parasitol 1966 52 660 663 10.2307/3276423 5969104

[B14] Contacos PG Coatney GR Orihel TC Collins WE Chin W Jeter MH Transmission of *Plasmodium schwetzi* from the chimpanzee to man by mosquito bite Am J Trop Med Hyg 1970 19 190 195 5443069 10.4269/ajtmh.1970.19.190

[B15] Deane LM Deane MP Ferreira NJ Studies on transmission of simian malaria and on a natural infection of man with *Plasmodium simium* in Brazil Bull World Health Organ 1966 35 805 808 5297817 PMC2476224

[B16] Collins WE *Plasmodium knowlesi*: a malaria parasite of monkeys and humans Annu Rev Entomol 2012 57 107 121 10.1146/annurev-ento-121510-133540 22149265

[B17] Wiwanitkit V Human knowlesi malaria and neurological complication: a new thing to be discussed in tropical neurology Acta Neurol Taiwan 2011 20 228 22009128

[B18] William T Menon J Rajahram G Chan L Ma G Donaldson S Khoo S Frederick C Jelip J Anstey NM Yeo TW Severe *Plasmodium knowlesi* malaria in a tertiary care hospital, Sabah, Malaysia Emerg Infect Dis 2011 17 1248 1255 10.3201/eid1707.101017 21762579 PMC3381373

[B19] Van den EP Van HN Van Overmeir C Vythilingam I Duc TN Hung LX Manh HN Anne J D’Alessandro U Erhart A Human *Plasmodium knowlesi* infections in young children in central Vietnam Malar J 2009 8 249 10.1186/1475-2875-8-249 19878553 PMC2773789

[B20] Berry A Iriart X Wilhelm N Valentin A Cassaing S Witkowski B Benoit-Vical F Menard S Olagnier D Fillaux J Sire S Le Coustumier A Magnaval JF Imported *Plasmodium knowlesi* malaria in a French tourist returning from Thailand Am J Trop Med Hyg 2011 84 535 538 10.4269/ajtmh.2011.10-0622 21460005 PMC3062444

[B21] Bronner U Divis PC Farnert A Singh B Swedish traveller with *Plasmodium knowlesi* malaria after visiting Malaysian Borneo Malar J 2009 8 15 10.1186/1475-2875-8-15 19146706 PMC2634766

[B22] Daneshvar C Davis TM Cox-Singh J Rafa’ee MZ Zakaria SK Divis PC Singh B Clinical and laboratory features of human Plasmodium knowlesi infection Clin Infect Dis 2009 49 852 860 10.1086/605439 19635025 PMC2843824

[B23] Hoosen A Shaw MT *Plasmodium knowlesi* in a traveller returning to New Zealand Travel Med Infect Dis 2011 9 144 148 10.1016/j.tmaid.2011.03.002 21481643

[B24] Kantele A Marti H Felger I Muller D Jokiranta TS Monkey malaria in a European traveler returning from Malaysia Emerg Infect Dis 2008 14 1434 1436 10.3201/eid1409.080170 18760013 PMC2603100

[B25] Coatney GR Collins WE Warren M Contacos PG The primate malarias Division of Parasitic Disease, producers 2003 10 Atlanta, GA: CDC original book published in 1971

[B26] Vythilingam I Noorazian YM Huat TC Jiram AI Yusri YM Azahari AH Norparina I Noorrain A Lokmanhakim S *Plasmodium knowlesi* in humans, macaques and mosquitoes in Peninsular Malaysia Parasit Vectors 2008 1 26 10.1186/1756-3305-1-26 18710577 PMC2531168

[B27] Snounou G Viriyakosol S Zhu XP Jarra W Pinheiro L do Rosario V Thaithong S Brown KN High sensitivity of detection of human malaria parasites by the use of nested polymerase chain reaction Mol Biochem Parasitol 1993 61 315 320 10.1016/0166-6851(93)90077-B 8264734

[B28] Rubio JM Post RJ van Leeuwen WM Henry MC Lindergard G Hommel M Alternative polymerase chain reaction method to identify *Plasmodium* species in human blood samples: the semi-nested multiplex malaria PCR (SnM-PCR) Trans R Soc Trop Med Hyg 2002 96 1 S199 S204 12055839 10.1016/s0035-9203(02)90077-5

[B29] Ta TH Ali-Tammam M Lanza M Rubio JM Merino L, Giusiano G Detection and identification of *Plasmodium* species by nested multiplex malaria PCR Manual of molecular methods for microbiological studies 2011 1 Buenos Aires: Asoc. Argentina de Microbiología 109 110

[B30] Van de Peer Y De Wachter R TREECON for Windows: a software package for the construction and drawing of evolutionary trees for the Microsoft Windows environment Comput Applic Biosci 1994 10 569 570 10.1093/bioinformatics/10.5.569 7828077

[B31] Thompson JD Higgins DG Gibson TJ CLUSTAL W Improving the sensitivity of progressive multiple sequence alignment through sequence weighting, position-specific gap penalties and weight matrix choice Nucleic Acids Res 1994 22 4673 4680 10.1093/nar/22.22.4673 7984417 PMC308517

[B32] DNA Oligos - modified & non-modified http://www.sigmaaldrich.com/configurator/servlet/DesignTool

[B33] Singh B Bobogare A Cox-Singh J Snounou G Abdullah MS Rahman HA A genus- and species-specific nested polymerase chain reaction malaria detection assay for epidemiologic studies Am J Trop Med Hyg 1999 60 687 692 10348249 10.4269/ajtmh.1999.60.687

[B34] Joveen-Neoh WF Chong KL Wong CM Lau TY Incidence of malaria in the interior division of Sabah, Malaysian Borneo, based on nested PCR J Parasitol Res 2011 12 104284 22013506 10.1155/2011/104284 PMC3195446

[B35] Siribal S Nakasiri S Looareesuwan S Chavalitshewinkoon-Petmitr P Identification of human malaria parasites and detection of mixed infection in Thai patients by nested PCR Southeast Asian J Trop Med Public Health 2004 35 5 9 15906626

[B36] Rahman KM Epidemiology of malaria in Malaysia Rev Infect Dis 1982 4 985 991 10.1093/clinids/4.5.985 6755616

[B37] Lim ES Current status of malaria in Malaysia Southeast Asian J Trop Med Public Health 1992 23 4 43 49 1364867

[B38] World Health Organization World Malaria Report 2012 2012 Geneva, Switzerland: WHO

[B39] Barber BE William T Grigg MJ Yeo TW Anstey NM Limitations of microscopy to differentiate *Plasmodium* species in a region co-endemic for *Plasmodium falciparum, Plasmodium vivax and Plasmodium knowlesi* Malar J 2013 12 8 10.1186/1475-2875-12-8 23294844 PMC3544591

